# Supplementary material for: DMRforPairs: identifying Differentially Methylated Regions between unique samples using array based methylation profiles
Source: BMC Bioinformatics. 2014 May 15;15:141. doi: 10.1186/1471-2105-15-141 (PMC4046028; doi:10.1186/1471-2105-15-141)
Supplement: Additional file 2 — DMRforPairs output for the comparison of A431-MCF7 and NA17018-NA17105. Please start from the HTML files in each folder. Available via the BMC Bioinformatics website. [file 1471-2105-15-141-S2.zip › 1394847754114233_MOESM2_ESM/A431_MCF7/figures/34511.pdf]

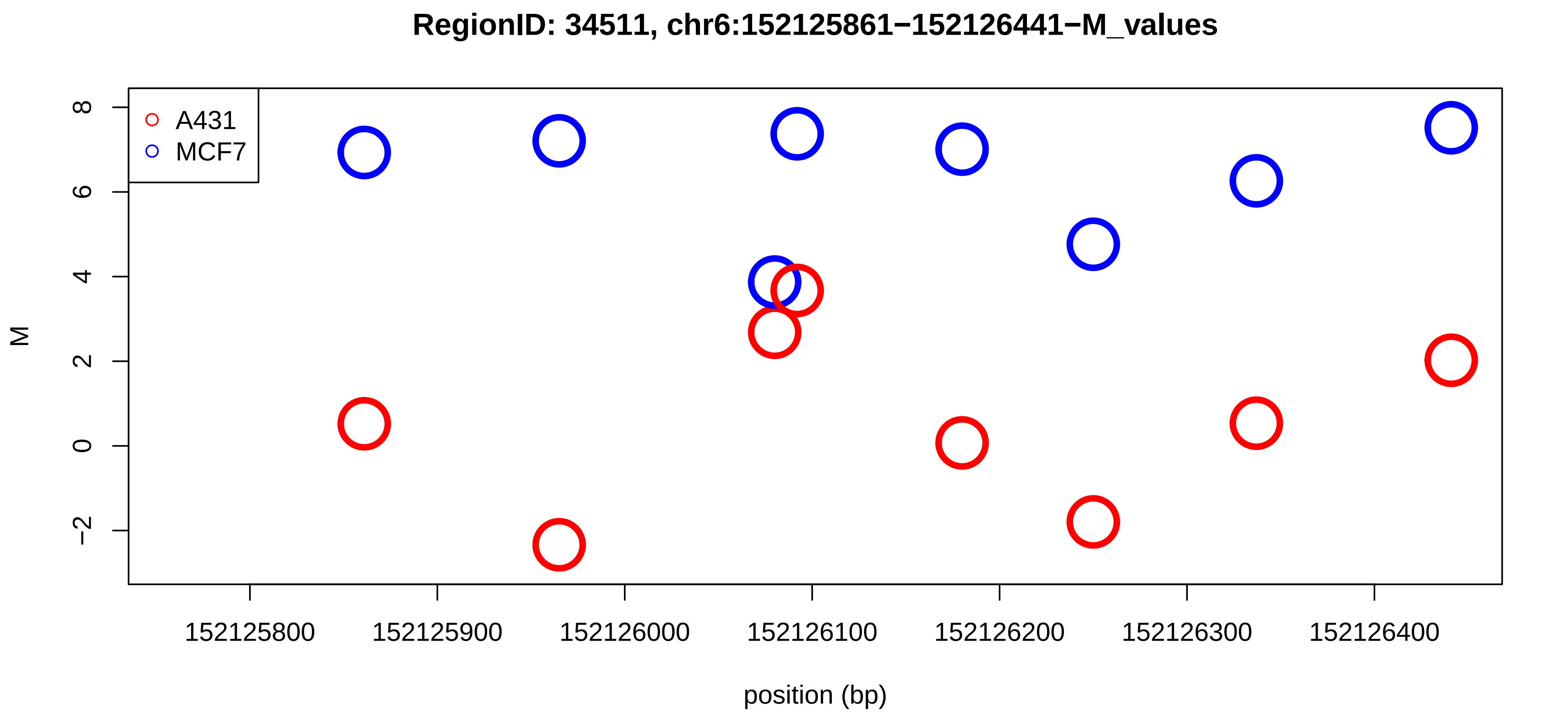
RegionID: 34511, chr6:152125861−152126441−M_values
A431
MCF7
M
−2
0
2
4
6
8
152125800
152125900
152126000
152126100
152126200
152126300
152126400
position (bp)

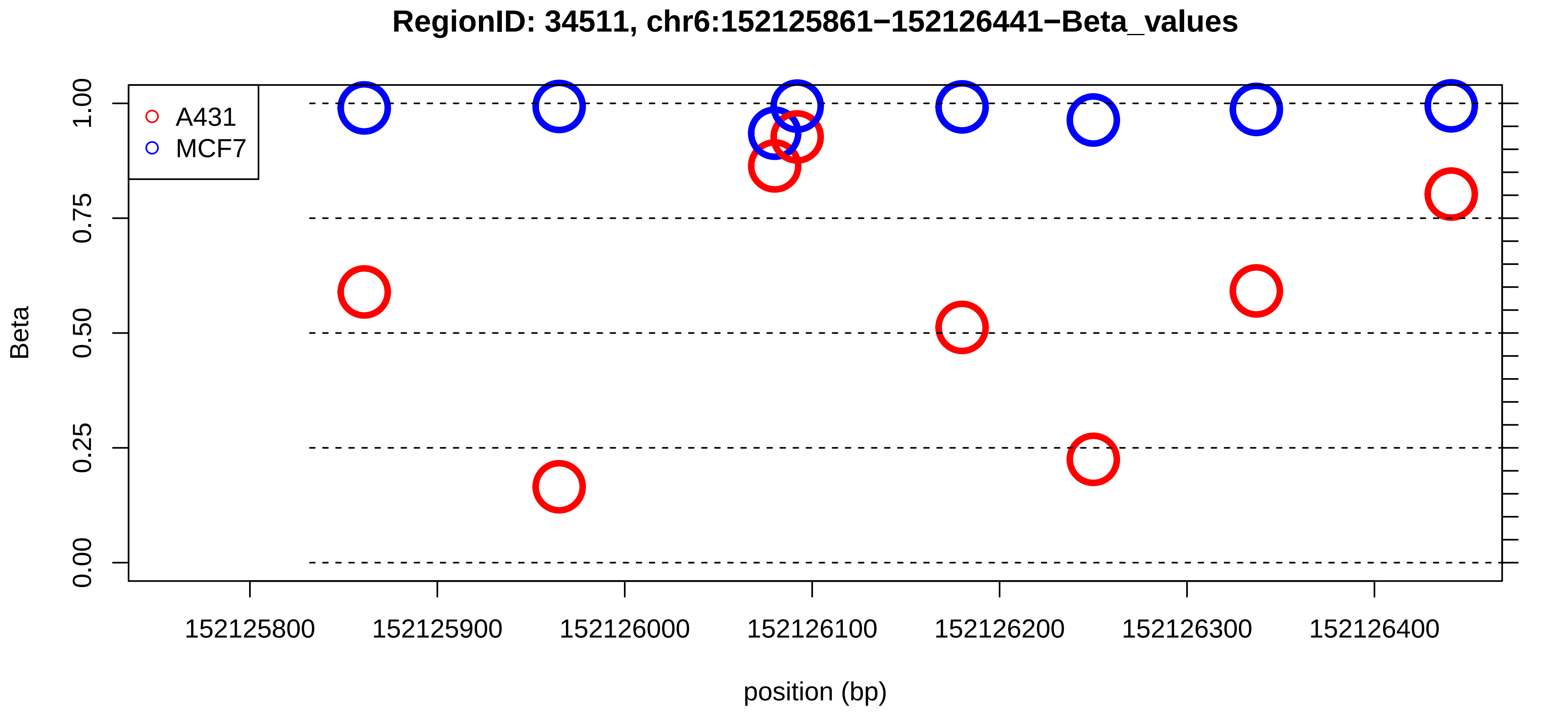
RegionID: 34511, chr6:152125861−152126441−Beta_values
A431
MCF7
Beta
0.00
0.25
0.50
0.75
1.00
152125800
152125900
152126000
152126100
152126200
152126300
152126400
position (bp)

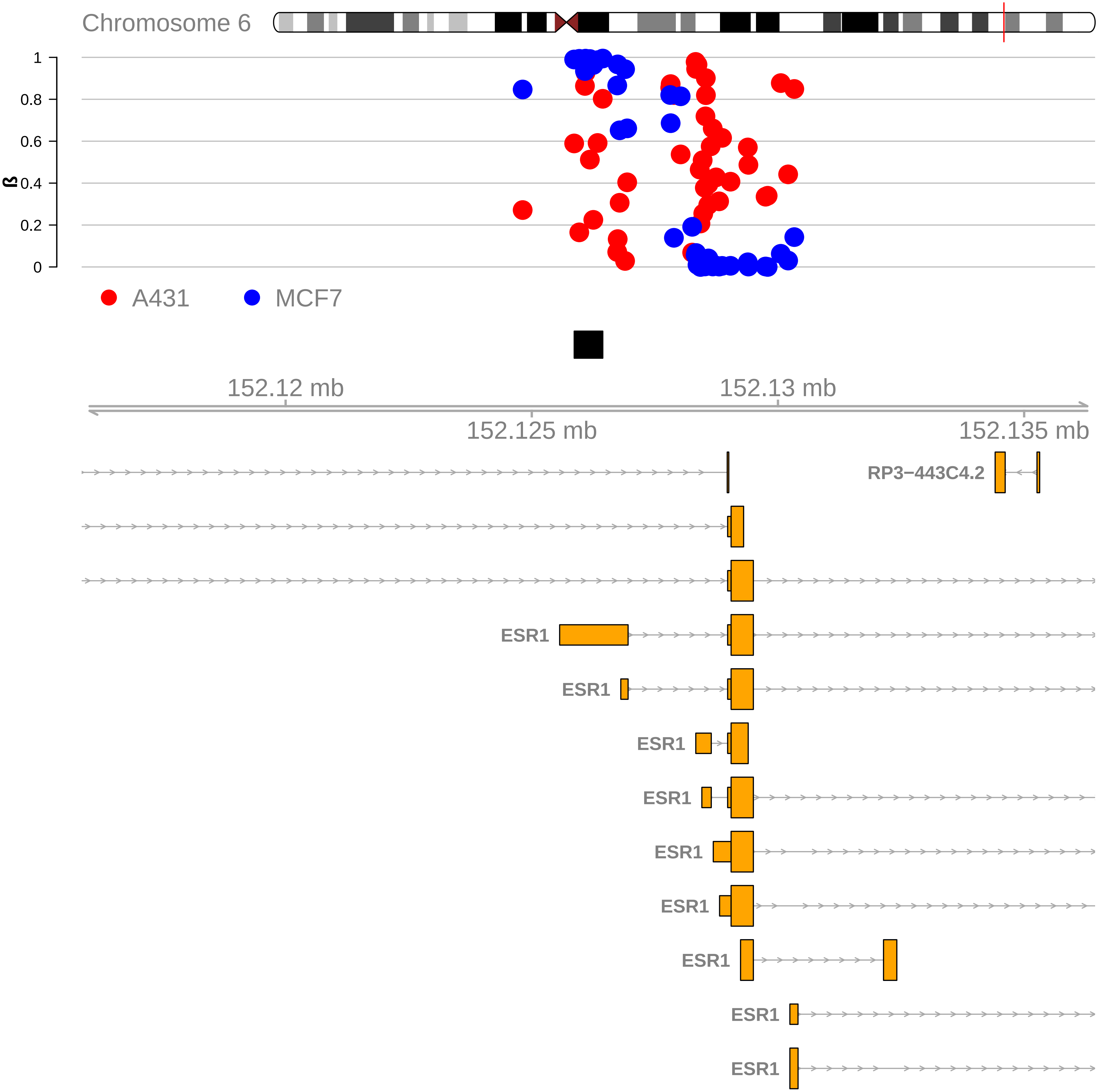

Chromosome 6
1
0.8
0.6
ß
0.4
0.2
0
A431
MCF7
152.12 mb
152.13 mb
152.125 mb
152.135 mb
RP3−443C4.2
ESR1
ESR1
ESR1
ESR1
ESR1
ESR1
ESR1
ESR1
ESR1
